# Quantifying Debris Flood Deposits in an Alaskan Fjord Using Multitemporal Digital Elevation Models

**DOI:** 10.3390/s21061966

**Published:** 2021-03-11

**Authors:** Matthew Balazs, Anupma Prakash, Gabriel Wolken

**Affiliations:** 1 Geophysical Institute, University of Alaska Fairbanks, 2156 Koyukuk Drive, Fairbanks, AK 99775, USA; aprakash@alaska.edu; 2 GeoNorth Information Systems, 561 East 36th Avenue Anchorage, AK 99503, USA; 3 Alaska Division of Geological & Geophysical Surveys, 3354 College Road, Fairbanks, AK 99709, USA; gabriel.wolken@alaska.gov

**Keywords:** DEM, SfM, DoD, LiDAR, dGNSS, sedimentation, debris flood, survey, Alaska, flood mitigation

## Abstract

Six DEMs over a 10-year period were used to estimate flood-related sedimentation in the Japanese Creek drainage located in Seward, Alaska. We analyze two existing LiDAR DEMs and one GNSS-derived DEM along with three additional DEMs that we generated using differential Global Navigation Satellite System (dGNSS) and Structure from Motion (SfM) techniques. Uncertainties in each DEM were accounted for, and a DEMs of Difference (DoD) technique was used to quantify the amount and pattern of sediment introduced, redistributed, or exiting the system. Through correlating the changes in sediment budget with rainfall data and flood events, the study demonstrates that the major flood events in 2006 and 2012—the 7th and 5th highest precipitation events on record—resulted in an increased sedimentation in the drainage as a whole. At a minimum the 2006 and 2012 events increased the sediment in the lower reaches by 70,100 and 53,900 cubic meters, respectively. The study shows that the DoD method and using multiple technologies to create DEMs is effective in quantifying the volumetric change and general spatial patterns of sediment redistribution between the acquisition of DEMs.

## 1. Introduction

Alluvial fans in recently deglaciated areas of the world, like the fjords of South-Central Alaska, are dynamic areas where the forces of water and gravity are actively at work and continuously reshape the landscape. They are also home to many people and often the ground on which infrastructure is built. During severe rainfall events, large quantities of sediment can be eroded from the surrounding slopes, become mobilized, and deposited into the lower reaches of the fjord in the matter of hours to days [1,2,3]. In a natural environment, this abundance of sediment may block the active channel of the alluvial fan and cause the water to avulse into another part of the fan, creating a new active water channel [4,5]. However, in cities located within fjords, the active river channel cannot be allowed to avulse as it would flood the developed areas of the fan and bring in unwanted sediment. To mitigate this from happening city planners incorporate various measures such as constructing levees, spillways, or diversionary tunnels to contain and direct the flow of water and the distribution of sediment [6,7]. These protective measures remain useful only as long as they have the capacity to hold and divert enough water and sediment. As the sediment in the protective corridors builds up, the infrastructure weakens and its capacity to redirect the water as planned is negatively impacted. To combat this, city managers and engineers must actively monitor and manage these protective areas. 

Deposition on alluvial fans occurs through a number of processes such as debris flows, debris floods, hyperconcentrated flows, clear-water floods, or other mass wasting events, such as avalanches, rockfall, and landslides [8,9]. Of these processes the debris flows and debris floods have the potential to bring in the most amount of sediment to an area in the shortest amount of time. The rheological differences between these processes can be summarized by the concentration of water and material that is being transported. Debris flows usually contain sediment load between 70–90% by weight and >50–60% by volume, whereas debris floods contain sediment concentration closer to 40–70% by weight and 40–50% by volume [10,11,12]. Recently, Church and Jakob [13] proposed a further classification within the category of debris floods based on their triggering mechanisms: meteorologically-generated debris floods, debris flow to debris flood dilution, and outbreak floods. 

Regardless if the primary mode of sedimentation is through debris flows or debris floods, the amount of material that is transported and deposited downstream will depend on the amount of available material in the channels and the amount of water that was present to move it [14,15]. Based on these two variables, watersheds can be classified as being transport-limited or supply-limited [16,17,18]. In a transport limited regime, there is an ample amount of sediment in the watershed, and the amount of material that is transported is dependent on the availability of water. Whereas in a bedrock watershed, that has little loose sediments to be moved, the volume that is transported will be limited to the amount of sediment, or supply, within the watershed. 

Recently deglaciated areas, such as the area of interest for this study, are transport-limited watersheds. The retreating glaciers that once filled the valleys have left behind vast deposits of till and glaciofluvial materials in the valleys upstream. Additionally, the unstable, over-steepened deglaciated slopes deliver a constant supply of sediment to the active channels through debris flows, rock and snow avalanches, and rockfall. The supply of easily mobilized sediment is also likely to increase through lateral debuttressing [19,20] and a warming climate causing increased weathering and erosion [21,22,23]. Given that there is a seemingly endless supply of material to be transported in these areas, there is a clear need for gaining a better understanding of how water, specifically large-magnitude rainfall events, will affect the sediment redistribution from the upper watershed to the alluvial fans. This is especially so within any areas of protective infrastructure. This research examines one such area, the flood control corridor of Japanese Creek located in the city of Seward in Alaska, which has a long history of debris floods. We use DEMs collected over a ten-year timespan to quantify the amount of sedimentation that occurred during two major rainstorm events. As there are no stream gauges or hydraulic models to inform us how water has affected the sedimentation, we compare the results to the historical meteorological data to determine what the sediment budget of the stream is in normal years, and what it is during flood events. This research lays the foundation to answer how much debris would be transported in storms of various magnitudes in this region; a question that could not be answered without the use of multitemporal DEMs we collected and generated. 

## 2. Overview of the Study Area

**Figure 1:**
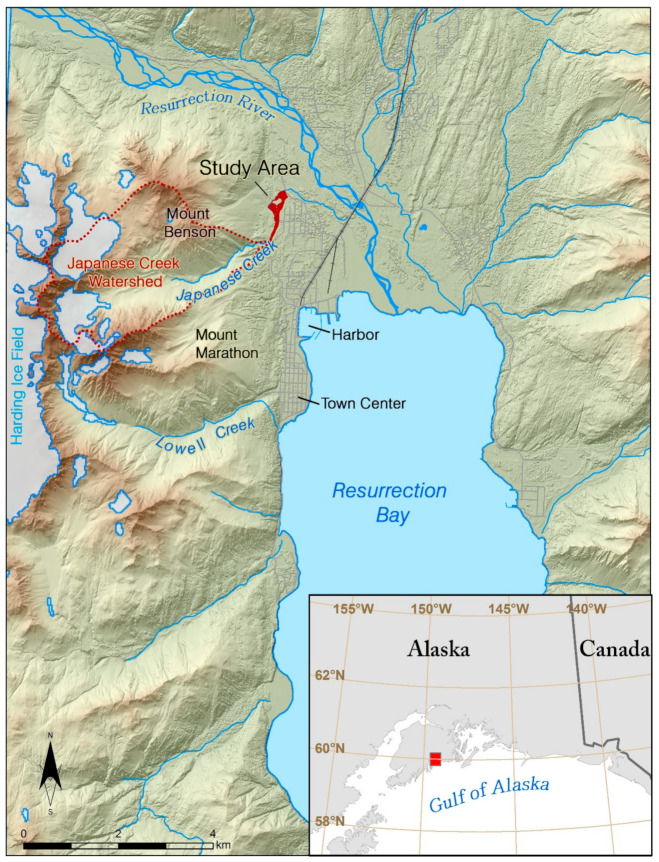
Map of the Resurrection Bay fjord, Seward, Alaska. Seward is located in the fjord-laden south-central coast of Alaska. The city is situated on alluvial fans created from the creeks originating from adjacent valleys, and the delta and flood plain of the Resurrection River. The area of interest for this study is the Japanese Creek reserved stream corridor (in red), which protects the northwest portions of the city from flood waters and debris flows originating from the Japanese Creek watershed to the west (outlined with a red dash line).

The city of Seward is nestled in the Resurrection Bay Fjord, located in the south-central portion of Alaska at approximately 60.10° N, 149.44° W (Figure 1). The city is constructed primarily on the alluvial fans and deltas of the streams and rivers that empty into the fjord’s water. Japanese Creek, located in the northwest portion of the city, drains a large and rugged watershed, and a significant portion of the city’s infrastructure is located on its alluvial fan. The catchment area is approximately 7.5 square kilometers of rugged and steep bedrock slopes which are comprised of mostly of shale and mudstones. The very fine- to medium-grained bedrock has been regionally metamorphosed to low greenschist facies during the latest Cretaceous to early Paleocene and is highly susceptible to weathering and erosion. [24,25]. 

The glaciers that filled the valleys have left behind abundant supplies of sediment which further erodes, transports, and deposits within the stream’s reaches. The sediment within the stream channel ranges greatly in size and shape, with large amounts of rounded to sub-rounded cobbles and boulders, interwoven with finer gravels, sands, and silt. In some locations angular and sub-angular cobbles and boulders can be found, likely introduced to the system from slope failures near the apex of the fan, outburst floods, or debris floods. 

The landcover within the catchment varies by elevation. At the highest elevations, slopes are comprised of exposed bedrock, colluvium, snow, ice, and few glaciers. At the mid-altitudes the slope is comprised mostly of exposed bedrock, till, perched lateral moraines, and colluvium. When soil is present in these areas, it is typically shallow and poorly developed where the vegetation, if present, are a mix of alpine herbaceous and shrub/scrub plants [26,27,28]. The lower parts of the slopes and the valley contain mostly Quaternary alluvial, colluvial, and glacial deposits. These are the only areas within the region that have significant soil development where large deciduous trees and Sitka Spruce can be found in addition to dense shrubs and some grasses. 

**Figure 2:**
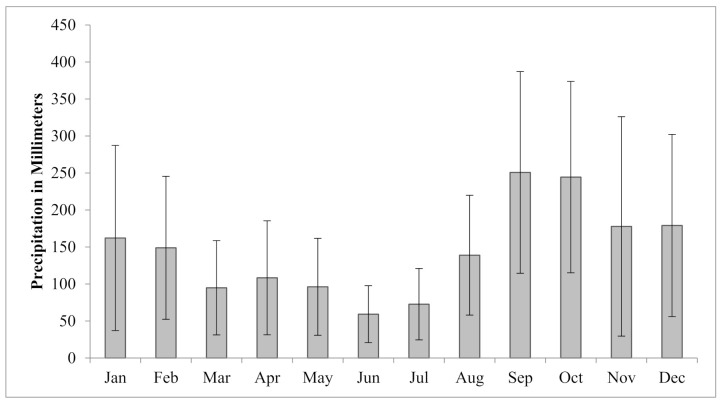
Average monthly precipitation for Seward Alaska, as measured at the Seward airport. The months of August–November periodically see heavy rains including remnants of tropical storms and typhoons which originate off the shores of Asia. Such storms have triggered both floods and slope failures throughout the area [31].

The steep topography and the associated orographic effect also create great variations in the weather. The mean annual precipitation ranges from 182 cm at the Seward airport in the valley (Figure 2), to more than 254 cm in the high-altitude glaciated areas surrounding the city [29,30]. The area also sees a higher monthly average rainfall during the months of September and October, and remnants from the Asian tropical storms or typhoons are not uncommon to reach the area in the months of August–November. 

**Figure 3:**
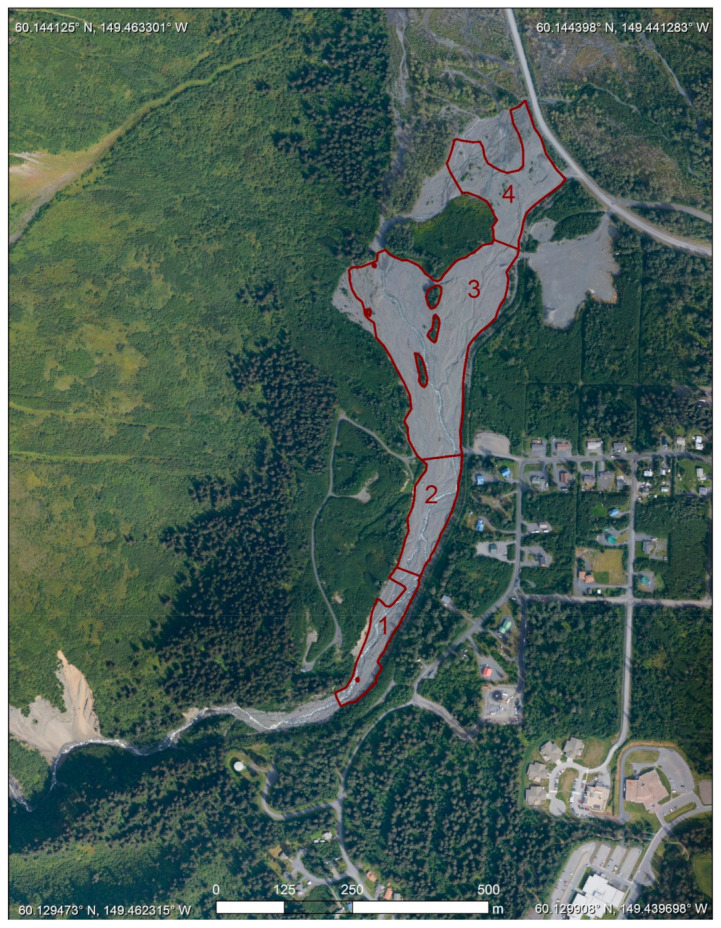
The Japanese Creek reserved stream corridor is broken up into four reaches. Reaches 1 and 2 are located upstream of the site of a previously existing bridge which was washed away in the flood of 2006 with 3 and 4 located downstream. The gaps in the area of analysis are due to the presence of vegetation, areas of unreliable data, or where data was not able to be collected. Of these areas, most are in areas that see little changes of sediment. As such, we only analyze the areas where all DEMs had valid elevation data.

The catchment of Japanese Creek exits through one pinch point between Mount Benson to the north and Mount Marathon to the south. This pinch point is the apex of the Japanese Creek alluvial fan and has historically been the location where outburst floods and debris floods have exited the catchment and caused damage to the community, most notably in 1966, 1969, 1976, 1982, and 1986 [30]. To protect properties built downslope of the apex the city replaced the existing earthen dams and constructed a more robust levee, which was completed in 2001 [32]. This levee serves to direct the flow of the stream sharply to the north. Downstream of the apex the stream is confined between the toe of Mount Benson to the west and the levee to the east where it flows at a gradient between 5 and 9 degrees which is typical of streams found on alluvial fans. This area, referred to as the Japanese Creek reserved stream corridor [33,34] and outlined in red (Figure 3), is the area of interest (AOI) for this study. 

**Figure 4:**
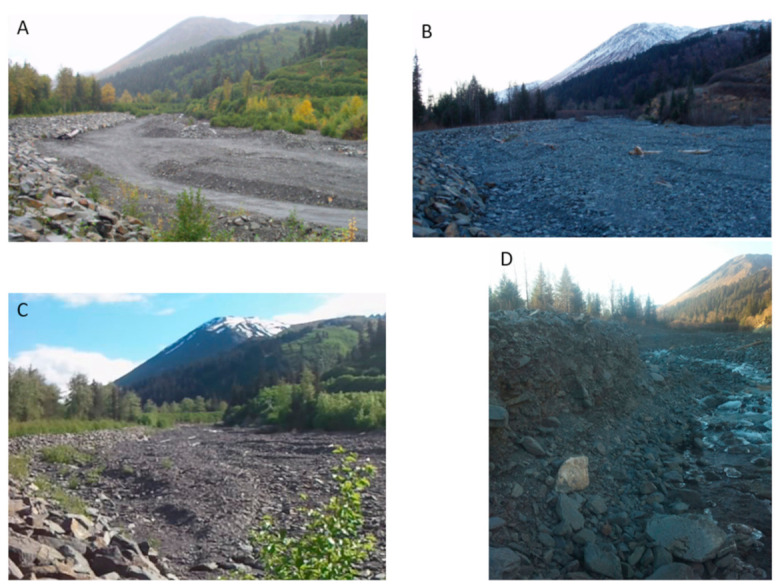
Photos taken at the boundary of reaches 2 and 3, looking upstream of the Japanese Creek Reserved stream corridor taken before and after to the severe storms of October 2006 and September 2012. (**A**) Photo taken just prior to the 2006 storm. Note the rip-rap levee on left [34]; (**B**) photo taken just after a severe storm in October 2006 showing vast amounts of sedimentation [34]; (**C**) photo taken in 2011 shows sediment redistribution and erosion that occurred during a period of typical rainfall events between 2006 and 2011; (**D**) photo taken in 2012, showing the upper reaches experiencing more erosion than sedimentation, compared to the storm in 2006. Photos A and B are reproduced with permission from Northwest Hydraulic Consultants, 2007.

The levee walls are made of large boulders and riprap on the downslope sides of creeks to divert the debris-laden flood waters away from, and to prevent avulsion from occurring in, critical areas (Figure 4). While the infrastructure is designed to constrain the flood waters of a 100-year flood event, it also constrains the area in which sediment can be deposited. During a flooding event the rapid increase in the amount of sediment in a corridor will drastically reduce the capacity to hold flood water. Overtopping of the levees and berms, or channel avulsion into other less restricted areas would devastate large parts of the community. To mitigate these issues, the local agencies and independent property owners have actively removed sediment from several reserved stream corridors, or relocated it within the corridor to build up berms or redistribute it to a less active section. While the strategy of restricting channel migration and sediment removal/redistribution has shown some success in mitigating the damage of floods, there remain questions about their long-term feasibility. 

A key variable in deciding how to maintain or add upon the existing infrastructure is knowing how much sediment is being deposited within the reserved stream corridors over time. It is also imperative to understand how less frequent debris floods will affect the sediment budget. As Japanese Creek is a transport-limited catchment, the amount of water is the main determining factor in the amount of sedimentation that will occur. Thus, to have a good understanding of the amount of sedimentation that should be expected in the reserved stream corridor, knowing the amount of water that will be present in the stream’s channels during a flood is needed.

Unfortunately, there are no in situ measurements of discharge rates for either normal water levels or times of flooding due to the absence of any stream gauges or other measurements. This leaves us to rely solely on meteorological measurements taken at the weather stations to establish a correlation between the magnitude of rainfall events and amounts of sedimentation that takes place. The few qualitative reports [32,35] and quantitative studies [30,36] that have been done in and near the study site show that intense or prolonged rainfall is the major factors of sediment mobilization in this area. There is also a large body of research that links precipitation thresholds to the triggering of debris flows and debris floods [37,38,39]. While these studies help gain a better understanding of when these events might be triggered, there has been no work in this area to relate the amount of sediment that may be mobilized with the magnitude of a precipitation event. To understand this relationship, we take a DEM-based approach to quantify the amount of sediment that has been deposited or eroded away during past debris floods and correlate these quantities with the available meteorological data. 

## 3. Data and Methods

Digital elevation models (DEMs) play a crucial role in determining how much sediment is being deposited within the reserved stream corridor and are often cited as the most important tools for modeling the risk of potential mass wasting events and hazard assessment studies [40,41,42,43]. Through differencing historic DEMs, a process referred to as DEMs of Difference (DoD), areas and volumetric amounts of sedimentation and erosion can be calculated for the interval between data collections of the DEM pair. 

A past study [34] estimated the amount of sediment delivered to the Japanese Creek reserved stream corridor in an October 2006 rainstorm by calculating changes in elevations between a LiDAR DEM that had been collected in January 2006 with a DEM they interpolated from a differential Global Navigation Satellite System (dGNSS) survey conducted in July of 2007. The study found that nearly 150,000 cubic meters of sediment was deposited within the reserved stream corridor during the debris flood. The sudden addition of sediment reduced the capacity of the corridor and increased the risk of flood waters overtopping the levee system. The authors concluded that the total volume of remaining space available in the stream corridor following the storm was merely 700,000 cubic meters, or 4.5 times the amount of sediment that was deposited in the storm of 2006. This study, while limited in scope by only examining one flooding event, illustrates the usefulness of using DEMs collected from very different survey means and techniques. 

Alaska has a dearth of high-resolution elevation data and there is sometimes a need to use any reliable data source available, as well as for opportunistic collection of new data when possible. Until recently, the foundational DEM data available for the State of Alaska was at 60-m resolution. The USGS’s 3D Elevation programs (3DEP) goal is to acquire complete high resolution (5 m) elevation coverage for the State by 2023 using InSAR technology as high-resolution LiDAR DEMs for such a vast and remote state is practically impossible [44]. Given such paucity of foundational DEM data we augment previously collected high-resolution elevation data through conducting additional surveys using dGNSS and Structure from Motion (SfM) techniques. The additional data were periodically collected in the field from 2011 to 2015 resulting in a total of seven high-resolution (approximately one meter) DEMs consisting of two bare-earth DEMs collected by LiDAR, four DEMs collected from GNSS points, and one digital surface model (DSMs) created from imagery (Table 1). 

The most commonly accepted definitions of DEMs, bare-earth DEMs, and DSMs draw distinctions between the three. DSMs contain the heights of vegetation, buildings, and all other objects for each pixel, while the bare-earth DEMs interpolate these areas to more closely match the elevation surface of the ground. However, in this study we were only interested in areas of the stream corridor that were unvegetated. The elevation values over these areas would not change between the types of the data and combining the bare-earth DEMs and the DSMs did not pose an issue. For this reason, we use the generic term DEM in the remainder of this paper. 

### 3.1. LiDAR

Two LiDAR datasets are available for the lower elevations of Resurrection Bay, including the study area. The city of Seward, the Seward Bear Creek Flood Service Area (SBCFSA), and the Kenai Peninsula Borough (KPB) commissioned Aero-Metric Inc. (Anchorage, AK, USA) to collect the first LiDAR survey in January of 2006. While the data were collected in winter, there was very little snow on the ground in the lower elevations and the little snow that was present is not thought to have affected the accuracy of the survey. During a subsequent dGNNS survey there were a total of 995 control survey points taken throughout the survey area and the vertical accuracy, assessed at the 95% confidence interval (1.96 × RMSE), was 0.10 m. The effective resolution of the DEM produced from this survey is one meter.

Following a moderately sized flooding event in the fall of 2009 the SBCFSA and the KPB commissioned Aero-Metric Inc. to fly another LiDAR survey in order to assess the damage of the surrounding area. A total of 754 GNSS surveyed points were used to assess the vertical accuracy, which at the 95% confidence interval was 0.09 m. The native effective resolution for this survey is 0.90 m and the data were resampled to 1 m to be in line with the other DEMs used in the analysis. 

We use the last return, or bare-earth products, of each LiDAR collection for this study as it represents the best available product for the computation of sediment change in this environment. Both surveys were published in the NAD83 Alaska State Plane Zone 4 (US Survey Feet) and the NAVD88 elevation were tied into the NGS benchmark “X 74” (PID: TT0396). We projected both DEMs into the NAD83 UTM Zone 6N and converted the elevation values to meters to be in line with the other datasets used in this study. There were some small areas within the AOI which had voids in the 2006 DEM which we chose not to close with a morphological filter or other means. 

### 3.2. GNSS Grid Surveys 

Four GNSS grid surveys were conducted within the reserved stream corridor of Japanese Creek. The first set was conducted in July 2007 by Northwest Hydraulic Consultants Inc. [34] with three additional surveys conducted by us in August 2011, October 2012, and September 2015. We used differential GNSS (dGNSS) systems with both real-time kinematic (RTK) and post-processing kinematic (PPK) methods, implementing both the GPS and GLONASS satellite constellations. All surveys were tied into the same NGS benchmark “X 74” (PID: TT0396) which was also used for the LiDAR surveys used in this study. However, setting a static base station over this benchmark was logistically not possible for the surveys so a “leap-frog” approach was used to survey in and establish a temporary benchmark nearer to the AOI where we then positioned the base station for the remainder of the survey. This setup minimized the gaps in radio communication between the GNSS base station and roving units caused by the surrounding steep topography and vegetation which is significant in the high latitude fjord environments. Establishing a permanent benchmark within the reserved stream corridor was not possible, so for each year we surveyed a new benchmark was established using these same methods. 

With the base station set up over a temporary benchmark the grid survey was conducted by securing the rover GNSS units to sturdy pack frames which were worn by crew members. Each rover was set to log its position every two seconds. A grid-like pattern was then walked throughout the AOI with the crew trying to maintain a one meter spacing between each transect. However, due to the very rugged terrain and the hazard of traversing an active glacially-fed creek, the route could not always be obtained systematically and the resulting grids were not uniform. In the worst cases we were not able to access small areas all together. Additionally, there were some portions where GNSS or radio communication to the base station was poor due to dense vegetation surrounding the perimeter of the AOI, the steep topography of the adjacent mountains, and possibly the high voltage electric wires that ran through a portion of the AOI. This led to areas of low point density or invalid survey data. To ensure that only the most reliable data were used we excluded any such points prior to the interpolation of the final DEMs.

Because of the irregularity in the grids we used the kriging statistical method [45] in the Geostatistical Analyst extension of ArcGIS software (ESRI ArcMap 10.2.1) to interpolate the DEM from the filtered surveyed points to create smooth DEM surfaces. The kriging resulted in a continuous surface. To ensure additional errors are not introduced into the final DoD calculation areas which were not surveyed, had poor GNSS signal, high error, or areas that had too low a point density were manually delineated and masked out from the final DEMs. Interpolating new elevation values in these masked areas over flat terrain may have been adequate. However, we erred on the side of being conservative and left them as voids in the DEMs throughout the AOI. Of final note, the 2011 survey was only conducted over reaches 1 and 2. While this collection lacks a significant portion of the AOI, we do use it in the analysis for the upper reaches, a critical portion of the alluvial fan. The dGNSS grid surveys of the AOI resulted in high spatial resolution DEMs (one meter) for each year and were projected into the NAD83 UTM Zone 6N datum.

### 3.3. Structure from Motion (SfM)

The final DEM used in this study was created by collecting aerial imagery and processing them using SfM algorithms [46,47]. 3493 aerial photos were acquired on 8 August 2015 at 3 s intervals at approximately 1260 m flying height above sea level. The flight was conducted close to solar noon and the flight lines were oriented north-south to minimize the differential illumination impact of bidirectional reflection distribution function (BRDF) that is more pronounced in the high latitudes [48,49]. The images had a 60% side lap and 80% end lap, resulting in a void-free coverage with an average ground sample distance (GSD) of 0.41 m. We processed the data using the Agisoft Photoscan software (Version 1.4.1, build 9525). The DEM was then resampled to one meter and projected into the NAD83 UTM Zone 6N datum to be consistent with the other datasets for the DoD analysis. 

The accuracy of a region-wide DEM was validated using dGNSS survey data collected at photo identifiable ground control points (GCPs) along the road systems of Seward, Alaska. These GCPs were collected in September of 2015 using two Trimble R8s GNSS receivers. The base station was tied into the NGS benchmark, “X-74” (PID: TT0396), located due west of the Seward Airport. We assessed the RMSE of the absolute vertical accuracy of this regional dataset to be 0.19 m. An additional survey was also conducted in August of 2015 within the Japanese Creek reserved stream corridor. This survey was used to validate the aerial survey within the study area and was not used in calculating the change in sedimentation. We collected a total of 1306 points which were surveyed while walking longitudinal transects of the reserved stream corridor and observed an absolute RMSE of 0.33 m between the two datasets. For the subsequent uncertainty analysis we used the more conservative assessment of 0.33 m in our calculations. 

### 3.4. DEMs of Difference (DoD)

While the main operation of computing a DoD is a simple subtraction of one DEM from another, much care is needed to ensure the best results. The workflow used here included several preprocessing steps before the DEMs were subjected to differencing. The preparation of the data was done using a combination of standard geoprocessing tools within ArcGIS and the Geomorphic Change Detection (GCD) add-on for ArcGIS [43]. Each DEM was first edited to void any areas where there were known issues in the survey data, such as where shadows affected the SfM construction or areas of very low accuracies in a dGNSS measurement. The DEMs were then intersected so that any areas that were voided out in one DEM would be voided in the rest of the DEMs. In our analysis we keep all the voids present in the data and only calculate change in the areas where valid elevation from all DEMs in the temporal stack are present. The notable exception to this is the 2011 survey, which only covers two out of the four reaches. We use this dataset to examine the sedimentation in the upper reaches only. The DEMs were then processed to be concurrent to one another (i.e., having the same extents, cell dimensions, orientation, and alignment). This was done to ensure there were no horizontal offsets between each DEM and additional resampling would not take place during the DoD operation. 

Next, the uncertainty of each DEM was assessed and areas where the uncertainty was high were voided or masked out of the final results. These uncertainties stem from several factors such as errors introduced from the sensor(s) and the environment, variance in the terrain, and the interpolation of the DEMs from the point data. To account for these factors, so-called error surfaces for each DEM were produced using the GCD toolkit in one of two ways. For data where the original point data are available (e.g., dGNSS and SfM data) a fuzzy inference system (FIS) was created which propagates the uncertainty based on point density of the survey, slope of the targeted terrain, and point quality of the survey data. In the case of the LiDAR data, where point data were not available, we use accuracies reported in the QAQC report or metadata that accompanied the data (reported above) to create an uncertainty layer of constant values for the respective datasets. The resulting error surfaces are used to mask out areas with high uncertainty so that they are not included in the final calculations in the DoDs. For example, if the calculated change of a given pixel is 0.25 m, but the propagated uncertainty of the two DEMs used in the calculation is 0.27 m, the pixel is masked out and not included in the final change measurements. 

Once the data were properly prepared, the DoDs were processed in two batches in order to create products that are easier to analyze and visually interpret. The first set compares all the DEMs to the 2006 data and treats 2006 as the baseline (e.g., 2007 minus 2006, 2009 minus 2006, 2011 minus 2006, etc.). The differences show how much sediment was gained or lost over the AOI since 2006, treating 2006 as the baseline. The second set created DoDs through differencing the DEMs in pairs along a series (e.g., 2007 minus 2006, 2009 minus 2007, 2011 minus 2009, etc.). 

### 3.5. Precipitation Data

**Figure 5:**
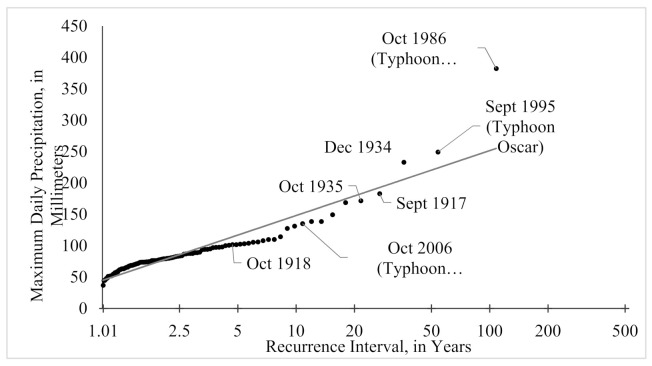
Recurrence Interval of maximum daily total precipitation in Seward for 1908–1925 and 1929–2018, measured in mm.

**Figure 6:**
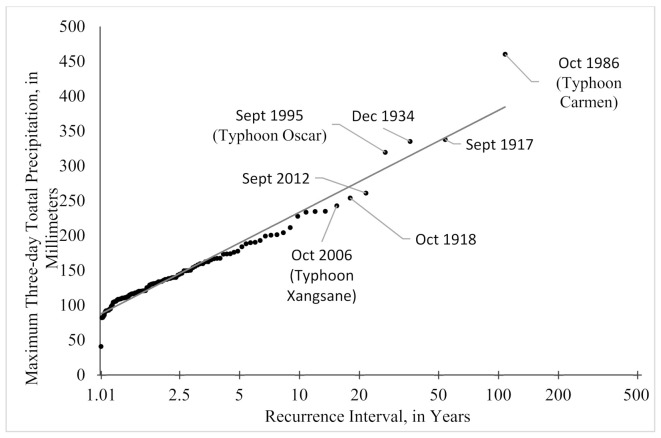
Recurrence interval of the maximum precipitation that occurred for each year in Seward. Some storms will have moderate rainfall but will stall over the region. To account for these types of events, the recurrence interval of severe rainfall events is estimated by summing three days of precipitation values. Computed from daily records, 1908–1925 and 1929–2018, measured in mm.

Data for the weather station located at the Seward airport were acquired for the period of 1908–1925, and 1929–2018 [31]. We calculated two recurrence intervals for this time period using the daily total precipitation (Figure 5) and the total precipitation for highest three-day period of each year (Figure 6). The procedure was adapted after others [50,51] who used multi-day sums to describe regional rainfall frequency.

While combining the three rainiest days of the year into a recurrence interval is not typical, we do so here to capture the signal of the long-lasting storms of more moderate daily total values. For example, there have been several years when the city experienced landslides and flooding after two to three days of sustained rains brought on by tropical storms (e.g., Typhoon Carmen, 1986; Typhoon Oscar, 1995; Typhoon Xangsane, 2006). While Typhoons Carmen and Oscar are captured in the top 10 events on both a one-day maximum precipitation and a three-day maximum precipitation plot, the advantage of evaluating the rainfall as a three-day sum can be illustrated with Typhoon Xangsane and other events such as the long-lasting storm in 1918. In the case of Typhoon Xangsane, the three-day event has a return interval of 15.4 years while the one-day event is 10.1 years and falls outside the top 10 most severe rainfall events (Figure 5 and Figure 6). The three-day period examined in 1918 further exemplifies these differences with a change of the return interval changing from 18 to 4.9 years when comparing three days to one. These return intervals are used to draw a correlation between the amount of sedimentation we observe, and the amount of water supplied for a given flood event. 

## 4. Results

**Figure 7:**
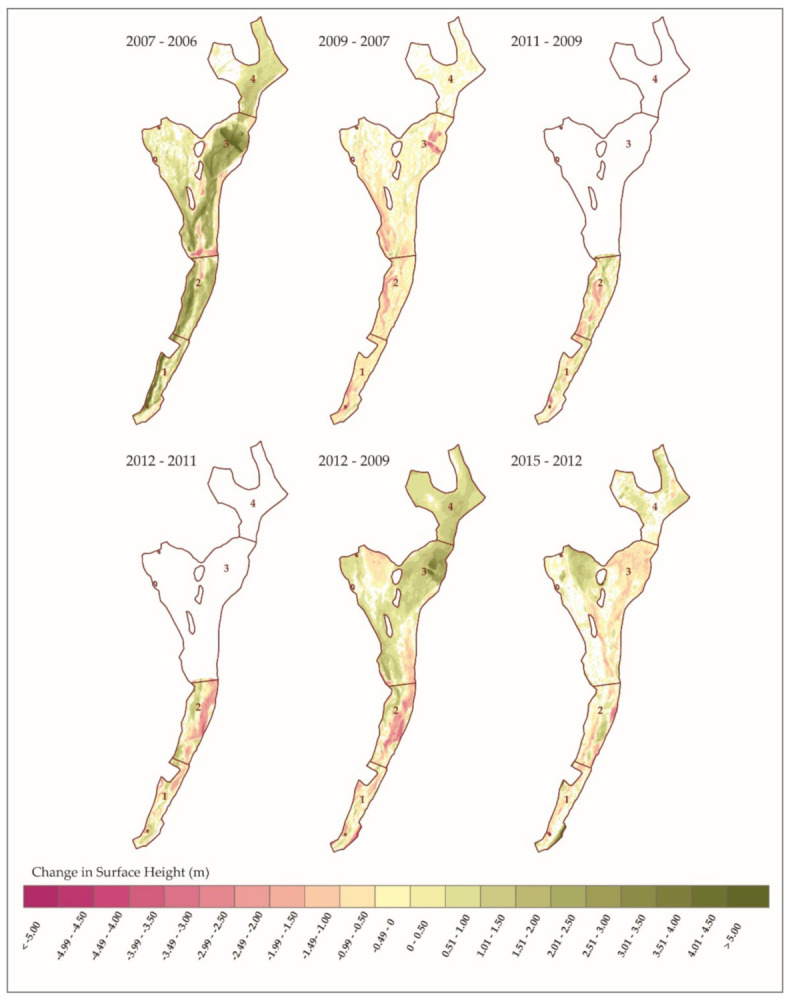
These DoDs were created by differencing the DEMs of successive surveys. The changes in the surface height are shown for each DEM pair, where the negative values denote the areas where erosion occurred, while positive values denote areas where deposition took place. The voids (white) are areas where the uncertainty of the measurement was higher than the calculated change.

The resulting DoDs show how both the quantity and patterns of sediment deposition and erosion has changed from year to year. As seen in Figure 7, areas where the calculated change was less than the level of the propagated uncertainty of the two DEMs are voided out of the final DoD. 

**Figure 8:**
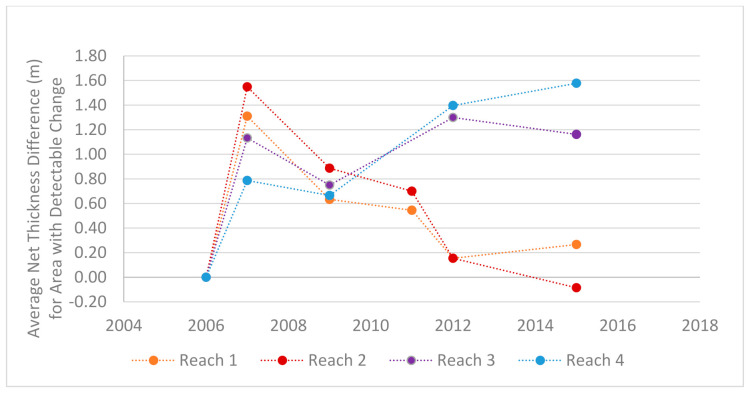
This graph shows the change in average thickness of sediments within the reserved stream corridor reaches. The values reported here show the increase in sediments from the baseline year of 2006, the first year with a high-resolution DEM. The additional sediment in the corridor following the storms of 2006 and 2012 can be seen. The sediment has continued to accumulate within reaches 3 and 4, while reaches 1 and 2 have returned to pre-2006 flood levels. Note: The 2011 survey was only conducted in reaches 1 and 2 and the volume in reaches 3 and 4 are unknown for that time interval.

From 2006 to 2015 the corridor as a whole gained approximately 100,400 cubic meters of sediment, most of which was deposited in the 2006 and 2012 storms. While most of the sedimentation occurred in the lower reaches the upper reaches also saw large amounts of sedimentation. At its peak the average net thickness of the sediments was 0.8–1.6 m across the four reaches (Figure 8). However, the normal regime of these reaches of the alluvial fan are erosional and transitional, and the sediment volumes are now back near the 2006 levels with having only an additional ~1200 cubic meters of sediment. This is important because it is this area which has the least capacity to hold sediment and it is closest to the apex which is of concern as the channel could avulse into the surrounding neighborhood if the channel was filled to the top of the levee.

**Figure 9:**
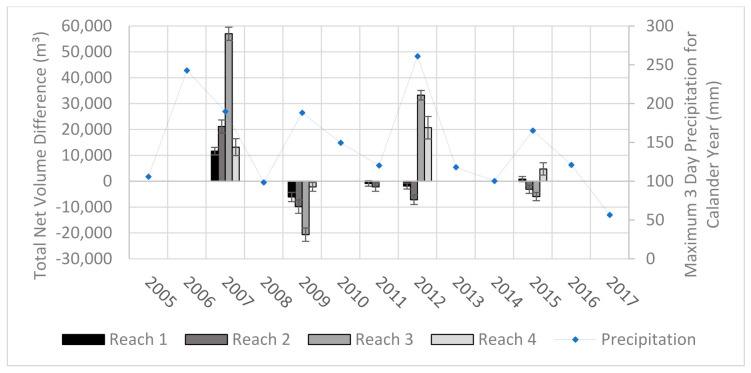
This graph shows the change in volume of the stream corridor and its associated reaches between acquisition years. The flood episodes of 2006 and 2012 clearly show strong but different depositional signals. The 2006 flood experienced significant deposition across all its reaches while the 2012 had a net increase in sediments only in reaches 3 and 4.

The obvious pattern that is shown in the DoDs is that the greatest amounts of change occur between the DEMs collected before and after a heavy rain event. The change between years of average rainfall is less drastic and have more areas voided out due to the detected change being less than the uncertainty of the input DEMs. When comparing this set with the rainfall records we can draw correlations between the amounts of sedimentation and the magnitude of the storms (Figure 9). While the elevation survey data were collected up to a year before a large precipitation event and up to a month after, there were no other processes that were capable of moving large amounts of sediment between each acquisition. Therefore, we attribute the amounts and movement of most of the sediment to the larger storms. The flood episodes of 2006 and 2012 clearly show strong but different depositional signals. The 2006 flood experienced significant deposition across all its reaches while the 2012 had a net increase in sediments only in reaches 3 and 4. While the record of changes over time is limited, these patterns of sediment change are to be somewhat expected as the lower reaches are the primary zones of deposition while the upper reaches are characterized by transitional processes. Reaches 1 and 2 are near the apex of the fan and are in a more tightly confined area than the lower reaches which spread out laterally and lessen in slope.

Table 2 illustrates the consequences of prolonged and severe rainfall. These events not only flood the area, but the floodwaters produced in very large events are often laden with debris. These debris floods have caused millions of dollars of damage and have plagued the city since its founding. Table 2 also illustrates the timing of the precipitation matters. The 3rd and 11th ranked three-day precipitation events, for example, were both in December where the precipitation fell as snow. While there were very likely localized avalanches, there were no known damages reported in the literature or city documents during this time.

## 5. Discussion 

### 5.1. Accounting for Uncertainties in Survey Measurements and DoD Calculations 

Understanding and accounting for uncertainty plays a critical role in this study. The data used in the analysis were acquired by a variety of techniques, used different sensors and equipment, and had unique uncertainties associated with the survey and subsequent interpolation of the point data into a DEM. The terrain that was surveyed was rugged, changed from year to year, and had steep river bars and banks that are challenging to survey and interpolate into a DEM. The timing of the surveys were sometimes years apart and there were long spans of time between the pre and post surveys of the 2006 and 2012 storm events. The changes in sedimentation that are due to the storms and changes which are due to normal erosion, transportation, and deposition are conflated in the DoDs. Finally, there is uncertainty in the amount of sediment that may have been removed from the AOI following the floods of 2006. 

Where possible we calculated uncertainty for a dataset using the raw point data collected from the survey. Where this was not possible, as with the LiDAR data, we relied on the published accuracies of the data in QAQC reports and metadata. Another dataset where uncertainty was not based on the raw data itself is the dGNSS surveys. Others have reported uncertainties ranging from 0.05–0.10 m using the same dGNSS survey techniques used here [52,53,54]. However, these past studies were conducted mostly on flat or sandy locations where the technicians would be able to maintain solid footing. The terrain in the Japanese Creek reserved stream corridor, by contrast, is filled with large cobbles and boulders and is very challenging to navigate in some locations, particularly in the steeper sections such as the banks of former stream channels. For these reasons we err on the conservative side to assign higher uncertainty values to the data rather than relying on the uncertainty values of each point collected at the time of the survey. The uncertainty of these points was set to a minimum of 0.20 m to account for any movement of the pack frame which the sensor was attached to and any uneven steps or stumbles that may have occurred during the traverse of the survey. 

The features within the survey site itself also effect the accuracy of the final DEMs. For example, the edges of the banks and bars of former channels are in general the steepest parts of our AOI. Much has been written about the relationship of slope and increased uncertainty in DEMs and how such areas should be taken into account in sediment budget estimates [55,56,57]. Another example of the feature within the landscape degrading the accuracy is the presence of tall and dense vegetation. These areas present two issues. The GNSS surveys are very difficult to conduct in these areas due to the loss of sufficient satellite coverage. These areas also have dark shadows in the imagery used in the SfM and are known to cause errors in the reconstruction of DEMs in places [58,59,60]. Both of these issues are accounted for by the use of the error surfaces and in some cases by manually introducing additional voids. 

The largest unknown in this study is the amount of sediment that may have been removed or redistributed within the reaches by the city following the severe rainfall event of 2006. We know that both the city and property owners removed some sediment following the flood but this quantity and the locations where it was taken from is unknown. Though the sediment removal operations have not been reported, we do know that the city and owners of the property have redistributed the sediment within reaches 3 and 4. Again, the specific locations of where the sediment has been redistributed is also unknown. For these reasons, our estimates of sedimentation are conservative and represent the minimum amount of sedimentation that would take place between the acquisitions of DEMs. 

### 5.2. Establishing a Link between Precipitation and the Amounts of Sedimentation

The DoDs clearly show a strong depositional signal following the storms of 2006 and 2012. However, the DEMs used to calculate the DoDs for these events were collected 18 and 14 months apart, respectively. The question remains as to how much of the change is due to those particular storm events and how much is due erosion and depositional rates typical of the stream? These questions have been cited several times by researchers and city managers alike for several decades [30,61] but until this study, estimates were not available due to the absence to elevation data which have sufficient spatial and temporal resolution. To help answer this question, we turn to the data collected in times of normal weather patterns and to the limited research of flooding events that have occurred in the area.

The DoDs show that in the absence of a major precipitation event the general trend in the sediment budget is that of increased erosion rather than deposition. This is generally the case for all four reaches with the exception of the 2012–2015 DoD where reach 4 and. to a lesser degree, reach 1 both experienced net deposition while the stream as a whole had a greater amount of erosion. The historic record also shows that the sedimentation that occurred in 2006 and 2012 are more typical during debris floods and outburst floods during heavy rain events which typically happen in the autumn months [30,32,35,61]. While the constant process of erosion, transportation, and deposition within the stream corridor will most certainly affect calculations in the DoD, the signal from the extreme debris floods dominates the time series. For these reasons the values reported here are defensibly conservative in nature. 

This study also begins the efforts of establishing a predictive correlation between the magnitude of a rain event and the amount of sediment deposited, but additional work must take place to adequately understand this relationship. For example, the amount of material that was deposited in the upper reaches during the storm of 2006 surpassed the amount deposited in 2012, even though both the single day and 3-day cumulative totals were greater in 2012. This could be explained if the flood in 2012 was closer to a clearwater flood than debris flood in sediment load. Assuming the sediment load was below 40% when the water exited the catchment and entered the alluvial fan, the additional amount of rainfall and larger volumes of flood water would increase the capacity of the stream. The increased hydraulic force would lead to more movement in the bedload within the upper reaches and more material would remain in suspension affectively redistributing the sediments from the upper reaches to the lower ones. During the 2012 flood event areas within reach 2 saw up to 3 m of erosion returning it close to pre-2006 levels.

### 5.3. Benefits of Using a Multi-Sensor Approach for DoD Studies 

There are clear advantages and disadvantages of using each technology outlined in this study. With the absence of a robust set of publicly-available high-resolution DEM data, the low-cost technologies of dGNNS and SfM make it practically feasible to acquire multiple elevation data sets over time with limited budgets. While the GNSS surveys were the most inexpensive techniques we used, they also required intense fieldwork. Additionally, the uncertainty of the point location was high since the antennas were attached to pack frames worn by people instead of being attached to a survey rod with a set height. The grids of these surveys were also not uniform and the density of points varied throughout the AOI due to the rugged terrain. In each survey year there were several locations that were deemed inaccessible due to the danger of crossing swift and deep glacially-fed waters. As such, the resulting accuracy also varies throughout the AOI which led to areas with low accuracies being masked out and interpolated or cut out of the analysis completely. 

In areas such as this study area it is not uncommon to have many days or weeks of rain or low altitude clouds that would prevent the acquisition of aerial photographs for SfM DEM creation. During these times, dGNNS surveys can provide researchers the data needed. Conversely, there are times where access to the study site might be impeded to a degree where no meaningful ground survey can be conducted. This may happen when a glacial stream is in a flood stage, or has changed course to block access by foot. In these situations aerial surveys, whether from LiDAR or SfM, may prove to be more timely and affective. The LiDAR surveys produced the highest quality data and resulted in the least number of voids. Unfortunately, it is often cost-prohibitive to collect LiDAR data. The SfM data also produced very high-resolution elevation models. It did, however, fail to produce accurate elevation values in areas of shadow and on some steep slopes. 

The remote and rugged regions of Alaska pose additional challenges for collecting high resolution DEMs. The high latitudes can lead to poor satellite coverage for dGNNS surveys which is exacerbated when local topography blocks additional satellites which are lower on the horizon. The low elevation of the sun also needs to be taken into account for the aerial surveys. Poor illumination and long shadows make DEMs difficult or impossible to be created through SfM techniques. It is because of the variety of environmental and logistical challenges that we recommend multiple technologies should be considered for investigating the potential impacts of geohazards, such as debris floods, on local infrastructure and hazard mitigation planning. While uncertainties can be in the range of several decimeters in some areas, we show here that these survey techniques are adequate for the applications outlined in this study.

## 6. Conclusions

This study provides valuable information that helps to reconstruct a history and pattern of sedimentation in the study area, which would not have been possible otherwise. The most significant finding was that there was an increased sediment load in the drainage as a whole and that major storms such as in 2006 and 2012 significantly decreased the sediment carrying capacity of the reserved stream corridor as a whole. The amount of sediment within the corridor decreases in years of normal precipitation and through human intervention, but the rate of this decrease is slow and the amount of sediment in the corridor is still well above where it was prior to 2006. If in the future, there is a series of storms of similar magnitudes that happen in close proximity to one another—two storms in a season, or one major storm two years in a row—it is possible the catchment will not have enough sediment-bearing capacity. This will put infrastructure and human lives at risk further down the slope. The results show that the techniques deployed in this study have the capability to objectively quantify the minimum amount of sediment deposited (or remaining) in the corridor between two snapshots in time. 

This research also lays the foundations for determining the long-term sedimentation trends and linking the amount of sedimentation that can take place in the Japanese Creek reserved stream corridor with precipitation amounts, factors that are significantly important for planning infrastructure development in the neighboring coastal city of Seward, Alaska. Until this study, there were insufficient data to make such a linkage in this area. Additional surveys over this area would provide more frequent snapshots of elevation that would facilitate a predictive modeling of sediment erosion and deposition pattern in different reaches as a function of the amount of precipitation. This same approach can be used in other glacial fjord areas where infrastructure is potentially threatened by significant sediment redistribution from local flooding and debris flows.

## Figures and Tables

**Table 1:** Digital elevation models used for landslide inventory mapping and sedimentation analyses. The 2015 survey was not used to calculate volumetric changes but served as ground truth to the aerial survey and SfM DEM produced in 2015. The 2011, 2012, and 2015 DEMs were generated for this research.

Year	Technology	Effective Spatial Resolution (m)	Agency ^1^	Comments
2006	LiDAR	1.0	SBCFSA/KPB	
2007	GNSS	1.0	NHC	
2009	LiDAR	0.9	SBCFSA/KPB	
2011	GNSS	1.0	UAF/DGGS	Covers Reaches 1&2 only
2012	GNSS	1.0	UAF/DGGS	
2015	SfM	0.41	UAF/DGGS	
2015	GNSS	1.5	UAF/DGGS	Used for accuracy assessment of SfM

^1^ Agencies: (SBCFSA) Seward Bear Creek Flood Service Area; (KPB) Kenai Peninsula Borough; (NHC) Northwest Hydrologic Consultants; (DGGS) State of Alaska Division of Geologic and Geophysical Surveys; (UAF) University of Alaska Fairbanks.

**Table 2:** Total three-day precipitation amounts with recurrence interval and known damage for Seward Alaska, 1908–2018. Dollar amounts are adjusted for inflation to 2018 values. Winter snow storm events are highlighted in grey.

Dates	Three Day Total Precipitation (mm)	Rank	Probability of Recurrence (%)	Recurrence Interval (Years)	Known Damages	Official Estimated Costs
9–11 October 1986	460.2	1	0.9	108.0	Severe widespread flooding, slope failures, debris floods, outburst floods	46 million
9–11 September 1917	337.9	2	1.9	54.0	Lowell Creek flood destroys school, railroad facilities, and private homes	1.9 million
4–6 December 1934	335.3	3	2.8	36.0	Unknown	
19–21 September 1995	319.5	4	3.7	27.0	Severe widespread flooding, slope failures, debris- floods, outburst floods	16 million
18–20 September 2012	261.1	5	4.6	21.6	Severe flooding, debris floods, failed levee, slope failures	
7–9 October 1918	254.0	6	5.6	18.0	Lowell Creek flood destroys hospital and private property	
8–10 October 2006	242.8	7	6.5	15.4	Localized floods in streams affecting subdivisions in area, debris floods, slope failures	11.2 million
1–3 October 1935	235.0	8	7.4	13.5	Lowell Creek: debris floods through historic downtown, severe damage to flume	
8–10 November 1908	234.6	9	8.3	12.0	Unknown	
11–13 October 1923	233.6	10	9.3	10.8	Unknown	
24–26 December 2001	227.8	11	10.2	9.8	Precipitation mostly snow, no damages reported	
10–12 September 1961	211.6	12	11.1	9.0	Localized floods in streams affecting subdivisions in area	
15–17 September 1938	204.2	13	12.0	8.3	Unknown	
22–24 October 2002	201.4	14	13.0	7.7	Localized floods in streams affecting subdivisions in area	14 million (Borough-wide)
25–27 August 1989	200.7	15	13.9	7.2	Damage to homes, roads, bridges, and infrastructure	11.2 million

## Data Availability

The data presented in this study are openly available through the Alaska Division of Geological & Geophysical Surveys at [https://doi.org/10.14509/29824] reference number [62].
